# A comparison of advertised versus actual cannabidiol (CBD) content of oils, aqueous tinctures, e-liquids and drinks purchased in the UK

**DOI:** 10.1186/s42238-023-00183-y

**Published:** 2023-07-13

**Authors:** Drusus A. Johnson, Megan Hogan, Ray Marriot, Liam M. Heaney, Stephen J. Bailey, Tom Clifford, Lewis J. James

**Affiliations:** 1 School of Sport, Exercise and Health Sciences, Loughborough University, Loughborough, Leicestershire UK; 2 Bridge Farm Group, Spalding, Lincolnshire UK

**Keywords:** Cannabidiol, Product label, Accuracy, United Kingdom

## Abstract

**Background:**

Cannabidiol (CBD)-containing products are sold widely in consumer stores, but concerns have been raised regarding their quality, with notable discrepancies between advertised and actual CBD content. Information is limited regarding how different types of CBD products may differ in their deviation from advertised CBD concentrations. Therefore, CBD concentrations were quantified and compared in aqueous tinctures, oils, e-liquids and drinks.

**Methods:**

Products (13 aqueous tinctures, 29 oils, 10 e-liquids and 11 drinks) were purchased online in the UK. CBD concentrations were quantified in aqueous tinctures, oils and e-liquids via high performance liquid chromatography (HPLC), and in drinks via gas chromatograhy-mass spectrometry (GC-MS).

**Results:**

Measured concentrations fell −25.7 ± 17.3, −6.1 ± 7.8, −6.9 ± 4.6 and − 0.03 ± 0.06 mg/mL below advertised concentrations for aqueous tinctures, oils, e-liquids and drinks, respectively (medians ± interquartile ranges; *p *< .05). Oils deviated relatively less (−19.0 ± 14.5%) from advertised concentrations than e-liquids (−29.2 ± 10.2%), aqueous tinctures (−51.4 ± 41.4%) and drinks (−65.6 ± 36.5%; *p* < .01), whilst e-liquids deviated less than aqueous tinctures and drinks (*p* < .05), and deviation was not different between aqueous tinctures and drinks (*p* = .19). Only 5/63 (8%) products had measured concentrations within 10% of advertised concentrations.

**Discussion:**

Similarly to previous studies, few products had measured CBD concentrations within 10% of advertised concentrations, with most falling below advertised concentrations. All individual product types deviated from advertised concentrations, with oils deviating least. These findings may be indicative of poor manufacturing standards, or that CBD undergoes degradation in consumer products. This reinforces concerns over quality of CBD-containing consumer products and may highlight the need for improved regulation of such products.

## Introduction

Cannabidiol (CBD) is one of over 100 phytocannabinoids present in the cannabis plant, but is unique in its availability as an ‘over the counter’ product in the UK, the USA and many European nations. Consumer demand for CBD has grown rapidly due to beliefs that it may help manage specific medical conditions, such as epilepsy, and/or improve outcomes relating to general health and wellbeing (Moltke and Hindocha 2021). Numerous types of CBD-containing products are now available, including oil- or aqueous-based tinctures, e-liquids, drinks, edibles, and topical creams/gels. However, analyses of CBD-containing products have raised concerns regarding their quality, with discrepancies between advertised and actual CBD content, and contamination with other, illicit cannabinoids (Grafinger et al. 2020; Gurley et al. 2020; Poklis et al. 2019; Liebling et al. 2020; Bonn-Miller et al. 2017; Pavlovic et al. 2018; Mazzetti et al. 2020). For example, of 29 CBD-oils purchased within the UK, only 11 (38%) fell within 10% of their advertised CBD content (Liebling et al. 2020), concurring with similar analyses from other nations (Grafinger et al. 2020; Gurley et al. 2020; Bonn-Miller et al. 2017; Pavlovic et al. 2018; Mazzetti et al. 2020). However, there is currently limited information regarding the extent to which various different types of CBD products deviate from their advertised CBD content, which would have important implications for informing consumer choices. Therefore, the present study assessed CBD concentrations in a broader range of products than has previously been examined within the UK, including oil- and aqueous-based tinctures, e-liquids and drinks.

## Methods

### Study design

Sixty-three products from 40 brands (13 aqueous tinctures, 29 oils, 10 e-liquids and 11 drinks) were analysed for CBD concentration. More products were not analysed due to monetary costs. Health food stores and online CBD specialists are the first and second most common locations to purchase CBD products in the UK (Community Research and 2CV 2019). Only one aqueous tincture and no e-liquids were found to be available from major UK-based health food retailers. Therefore, aqueous tinctures and e-liquids were purchased online after searching keywords such as ‘CBD aqueous drops’ and ‘CBD vape’; the first 13 and 10 suitable products found, respectively, were purchased. For each aqueous tincture and e-liquid identified, an oil product was purchased from the same brand at the most similar advertised CBD concentration available (no oil was available to match one aqueous tincture). This enabled the comparison of oils *versus *e-liquids and aqueous tinctures, whilst potentially eliminating different companies’ manufacturing standards as a confounding factor. Because most aqueous tinctures, e-liquids and their paired oils were purchased from online retailers, additional oil products were purchased online from major UK health food retailers to provide a more representative sample of oils purchased by UK consumers (Community Research and 2CV 2019). All drinks were purchased online from major UK health food retailers. Products were analysed in a blinded fashion.

### Product analysis

CBD and cannabidiolic acid (CBDA) concentrations were quantified in aqueous tinctures, oils and e-liquids via high performance liquid chromatography (HPLC) with a Raptor ARC-18 LC reverse phase column (2.7 μm, 150 mm × 4.6 mm; Restek, PA, USA) using ultraviolet detection (228 nm). Reported CBD concentrations using HPLC reflect the combination of measured CBD and theoretic conversion of CBDA to CBD (Wang et al. 2016). Samples were analysed in singular except for a random subsample of nine products, analysed in duplicate; mean coefficient of variation = 0.17%. CBD concentrations were quantified in drinks via gas chromatography-mass spectrometry (GC-MS) using an Agilent 7890A (CA, USA) Gas Chromatograph coupled to an Agilent 5975C Mass Spectrometer, with an HP-5MS column (30 m, 0.25 mm, 0.25 μm). CBD concentrations quantified using GC-MS represent combined CBD and CBDA, as CBDA is decarboxylated upon injection into the gas chromatograph. All concentrations were calculated by comparing the peak area of a cannabinoid standard of known concentration (Cerilliant®, TX, USA) to the samples. HPLC and GC-MS analysis conditions are displayed in Table 1.

Cannabinoids were extracted by diluting products to 1 mg/mL for HPLC analysis, or 0.001 mg/mL for GC-MS analysis, based on advertised CBD concentrations. Oils, e-liquids and highly viscous aqueous tinctures were diluted mass/mass in 50:50 isopropyl alcohol (IPA):methanol, except for two products that failed to fully dissolve in IPA:methanol, so were diluted in 100% IPA. Drinks and other aqueous solutions were diluted mass/volume in 100% methanol, then homogenised with an ultrasonic homogeniser (20 W; 50% amplitude; H7 probe) and the supernatant filtered (0.22 μm). Products for HPLC analysis were then diluted a further 10-fold in methanol (i.e., to 0.1 mg/mL, based on advertised concentrations). The HPLC assay precision is ≤5.5% for CBD and ≤4.6% for CBDA. The GC-MS assay precision is ≤3.6%. The HPLC assay accuracy is 95–112% (0.001–0.15 mg/mL) for CBD and 99–102% (0.001–0.005) for CBDA. The GC-MS assay accuracy is 97–116% (0.0001–0.001 mg/mL). GC-MS was applied to drinks as this method has a lower quantifiable range, outlined above, which was necessary to quantifiy the lower CBD concentrations in drinks *versus*. other product types (the quantifiable concentration ranges do not correspond to reported CBD product concentrations as products were diluted during cannabinoid extraction). The pH of drinks was measured using a SciQuip pH meter (PHS-25CW/3BW, Newtown, UK), calibrated with pH 4.00 and 7.00 solution. The electrode was rinsed with deionised water between samples.

**Table 1:** HPLC and GC-MS settings for quantification of cannabidiol (CBD) concentrations

Parameter	Specification
HPLC column temperature	30°C
HPLC flow rate	1.5 mL/min
HPLC analysis time	10 min
HPLC injection volume	5 µL
HPLC mobile phase conditions	Mobile phase A: aqueous ammonium formate (5mM)/formic acid (0.1%) Mobile phase B: acetonitrile/formic acid (0.1%) Isocratic, 25% mobile phase A
GC-MS carrier gas	Hydrogen at 1 mL/min
GC-MS inlet temperature	200°C
GC-MS injection volume:	1 µL at 50:1 split ratio
GC-MS temperature programme	60°C and held for 2 min, increasing to 300°C at 8°C/min and held for 10 min
GC-MS ion source temperature	230°C
GC-MS mass analyzer temperature	150°C
GC-MS analysis time	42 min
GC-MS scan range	40–600 m/z at 1 Hz

*HPLC *High-performance liquid chromatography, *GC-MS *Gas chromatography-mass spectrometry

### Statistical analysis

Advertised and measured CBD concentrations were compared for each product type using Wilcoxon signed-rank tests due to non-normal data distributions (Shapiro-Wilk test *p *< .05 and histograms shaped non-normally). Relative discrepancies between advertised and actual concentrations were compared between product types using one-way ANOVAs for unequal variance on rank-transformed scores (Zimmerman and Zumbo 1993). If significant, post hoc comparisons were made using *t*-tests for unequal variance on rank-transformed scores with a Holm-Bonferroni correction (Zimmerman and Zumbo 1993). This approach was selected due to unequal sample sizes and violations to both normality and homogeneity of variance (Levene’s test on medians*p* < .05). Comparisons between oils and brand-matched aqueous tinctures and e-liquids were made using an independent samples *t-*test or Mann-Whitney *U* test, depending on data normality. Relationships between variables were examined using Spearman’s ranks or Pearson’s correlations, depending on data normality. All statistical tests were two-tailed, significance accepted at *p* < .05, performed in SPSS-25 (IBM, NY, USA).

## Results

**Fig. 1:**
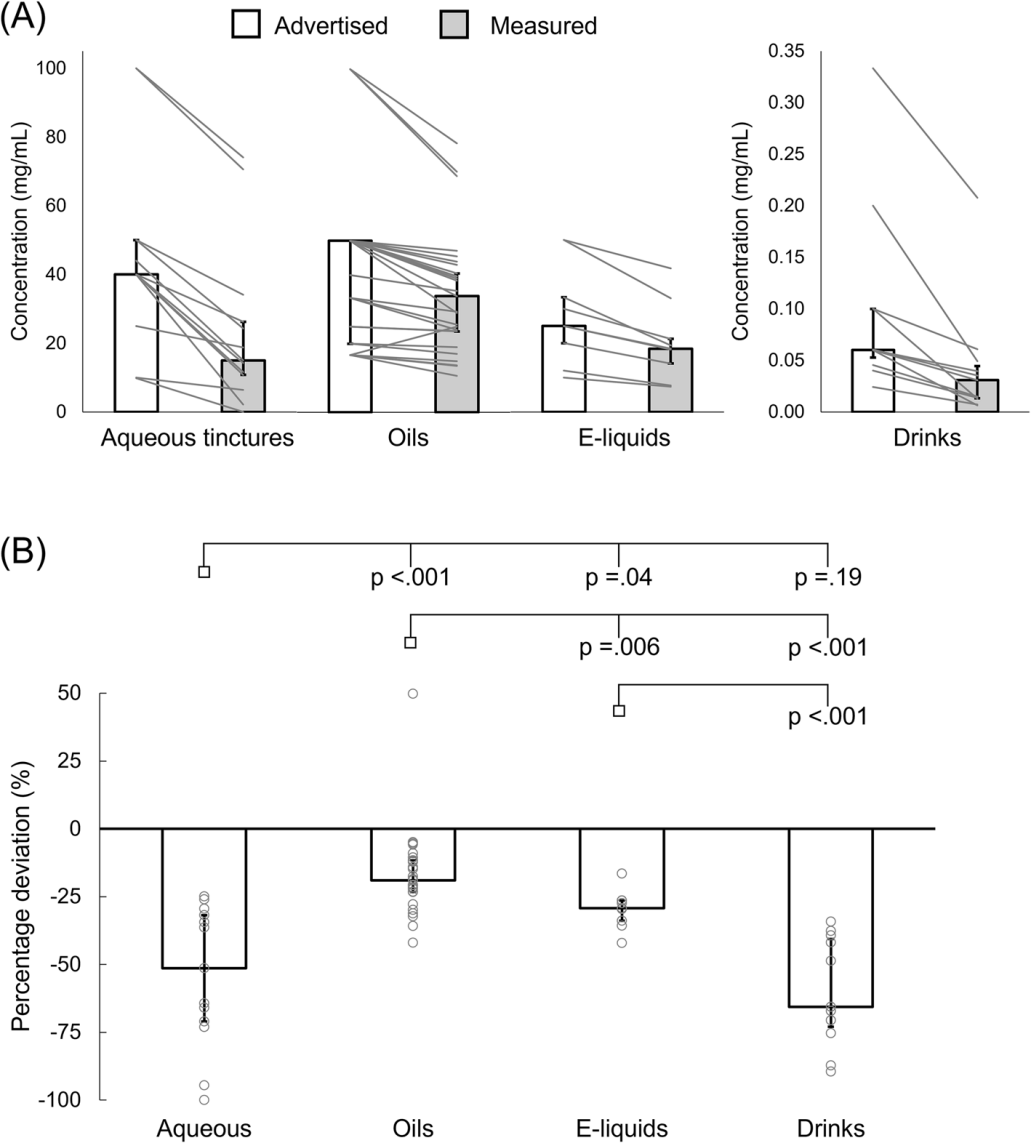
Absolute and relative deviation from advertised cannabidiol (CBD) concentrations for each product type. **A** Advertised *versus* measured absolute cannabidiol concentrations (*p* < .001 for each product type). **B**  Relative deviation from advertised cannabidiol concentrations; columns and error bars are medians ± interquartile ranges; grey lines (**A**) and open circles (**B**) represent individual products; *p*-values on panel (**B**) correspond to pairwise comparisons between the condition below an open square *versus* the condition under each branch stemming from that square (*p *< .001 for ANOVA main effect)

Measured CBD concentrations fell −25.7 ± 17.3, −6.1 ± 7.8, −6.9 ± 4.6 and −0.03 ± 0.06 mg/mL below advertised concentrations for aqueous tinctures, oils, e-liquids and drinks, respectively (median ± interquartile range [IQR]; *p* < .001 for all products; Fig. 1). Only 5/29 oils had measured concentrations within 10% of that advertised, and all other products fell >10% below advertised, except for a single oil that was 50% greater than advertised (Fig. 1). Relative deviation from advertised concentrations differed depending on product type (*p* < .001 for ANOVA main effect) and was less for oils *versus* all other product types (*p* < .01 for all comparisons), less for e-liquids *versus* aqueous tinctures (*p* = .04) and drinks (*p* < .01), but was not different between aqueous tinctures and drinks (*p* = .19; Fig. 1). Median (± IQR) relative deviations from advertised concentrations were −51.4 ± 41.4%, −19.0 ± 14.5%, −29.2 ± 10.2%, and −65.6 ± 36.5% for aqueous tinctures, oils, e-liquids and drinks, respectively (Fig. 1). Median (± IQR) measured and advertised CBD concentrations for the entire dataset were 19.2 ± 30.7 mg/mL and 33.3 ± 33.3 mg/mL, respectively (median difference: −6.6 ± 12.3 mg/mL [−29.5 ± 23.1%]; *p *< .001). Mean (± standard deviation [SD]) pH of drinks was 4.0 ± 0.8.

When comparing brand-matched oils and aqueous tinctures, absolute and relative discrepancies between measured and actual CBD concentrations were (mean ± SD) −12.7 ± 10.2 mg/mL and −20.4 ± 10.9% for oils, *versus* −24.3 ± 8.9 mg/mL and −51.5 ± 23.7% for aqueous tinctures. Relative discrepancies were greater for aqueous tinctures (*p* = .01). For brand matched oils and e-liquids, absolute discrepancies between advertised and measured CBD concentrations were (median ± IQR) −4.6 ± 9.0 mg/mL and −6.6 ± 6.3 mg/mL. When comparing relative discrepancies, deviation from advertised concentration (mean ± SD) was less for oils (−19.1 ± 11.9%) than e-liquids (−31.0 ± 7.7%; *p* = .03).

**Fig. 2:**
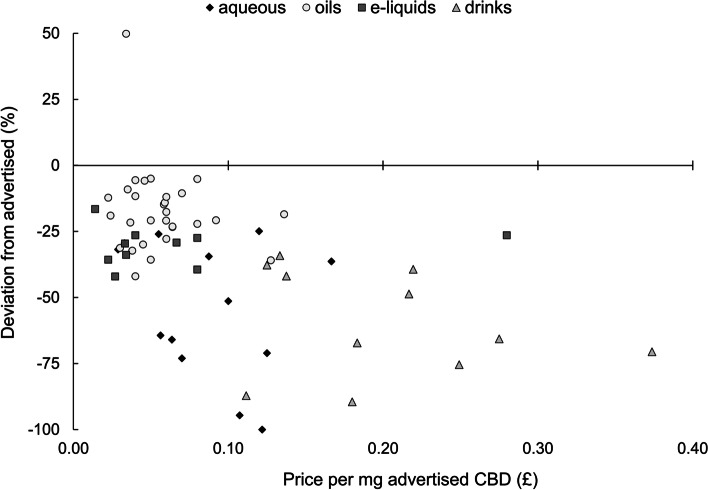
Relationships between product price (normalised to advertised CBD concentration) and relative deviation from advertised CBD concentrations for each product type. Correlation coefficients are as follows: all products (*R*=−.48, *p* < .001), aqueous tinctures (*R *= −.26; *p* = .39), oils (*R*_S _= −0.10; *p* = .60), e-liquids (*R*_S _= 0.11; *p* = .76) and drinks (*R *= −.24; *p* = .47); *R* = Pearson correlation; *R*_S_ = Spearman’s rank

There was no linear relationship between price (normalised to advertised CBD concentration) and relative discrepancy between measured and advertised CBD concentration for aqueous tinctures (*R *= −.26), oils (*R *= −.10), e-liquids (*R* = .11) or drinks (*R *= −.24; *p* > .05 for all products; Fig. 2). These variables demonstrated a negative relationship when examining all products together (*R *= −.48, *p* < .001), but this was driven by systematic differences between product types, e.g., oils deviating less than drinks, whilst simultaneously costing less per mg CBD.

## Discussion

Measured CBD concentrations of a broad range of UK-based products fell, on average, substantially below what was advertised, with only 5/63 (8%) products within 10% of advertised concentrations. The median deviations from advertised concentrations (−66 to −19%) were substantially greater than the measurement error associated with HPLC and GC-MS analysis, reinforcing concerns surrounding CBD products within the UK, whereby CBD consumers may consistently fail to achieve their desired CBD dose. Furthermore, the likelihood of achieving a desired dose may be dependent on product type, with oils deviating least from advertised concentrations. However, observed deviation is seemingly independent of product price.

The proportion of products measured within 10% of advertised concentration (8%) is at the lower end of previous analyses, in which ~ 10–40% of products have met this target (Grafinger et al. 2020; Liebling et al. 2020; Bonn-Miller et al. 2017; Pavlovic et al. 2018; Mazzetti et al. 2020), including 38% in a UK-based analysis of 29 oils (Liebling et al. 2020). The greater deviation from advertised concentrations in e-liquids, aqueous tinctures and drinks, which have more rarely or never been included in such analyses, reduced the proportion of products meeting this cut-off, which was 17% (5/29) for oils. Systematic differences in analytical techniques may also contribute to some minor variation between studies. Nevertheless, available evidence consistently suggests that only a minority of products contain CBD concentrations within acceptable limits of what is advertised.

The lesser deviation from advertised concentrations by oils in this study supports Bonn-Miller et al. (Bonn-Miller et al. 2017), who observed that oils were less frequently mislabelled than e-liquids in a sample of US products (22/40 *versus *21/24). Such findings may help inform consumer choice when purchasing CBD products. However, without repeat analyses, it is unclear how much CBD concentrations vary between batches of individual products, and so whether products that deviate more/less than others do so consistently. Available market research data suggest poor consumer awareness regarding CBD product quality. The consistent over-labelling of CBD concentrations in products is contradictory to findings that most UK consumers believe that the CBD product(s) they purchase are ‘high quality’ and contain CBD concentrations equivalent to what is advertised (Community Research and 2CV 2019). Despite this, perceived product quality is the primary factor in determining consumer choice of CBD product (Community Research and 2CV 2019).

It is unclear whether deviations from advertised CBD concentrations are due to discrepancy at the point of manufacture, or degradation afterwards. CBD in e-liquids can degrade 15–20% over 30 days when exposed to natural light or constant 37°C temperature (Mazzetti et al. 2020), suggesting that degradation may contribute to the discrepancies observed. Furthermore, variable rates of degradation in different solutions (Sørensen and Hasselstrøm 2018; Kosović et al. 2021) could potentially contribute to differences observed between product types. The lesser discrepancies observed in oils *versus* brand-matched e-liquids and aqueous tinctures indicate that this may be the case, as manufacturing standards would likely be comparable between products of the same seller. The tendency for greater discrepancies in aqueous products may suggest that CBD is more unstable when dissolved in water than in oils or substances such as propylene glycol, while the acidic pH of drinks may have exacerbated degradation in those products (Yangsud et al. 2021).

## Conclusions

The over-labelling of CBD concentrations within UK products highlights the need for improved product standards, which may necessitate clearer legislative guidance on acceptable tolerance limits for advertised CBD concentrations. The magnitude of deviation from advertised CBD concentrations differed between product types but was not related to product price, with CBD-oils deviating less than aqueous tinctures, e-liquids and drinks. Future research may seek to determine rates of CBD degradation in consumer products, and within-product variability in labelling accuracy. Furthermore, CBD used in research investigating its psychological or physiological affects should be analysed to confirm that CBD concentrations are appropriate.

## Data Availability

The full dataset is publicly available at 10.17028/rd.lboro.16920004.

## Abbreviations

CBD: Cannabidiol
CBDA: Cannabidiolic acid
HPLC: High-performance liquid chromatography
GC-MS: Gas chromatography-mass spectrometry
IPA: Isopropyl alcohol
SD: Standard deviation
IQR: Interquartile range
